# Adult onset lung disease following transient disruption of fetal stretch-induced differentiation

**DOI:** 10.1186/1465-9921-10-34

**Published:** 2009-05-06

**Authors:** Joseph J Hudak, Erin Killeen, Ashok Chandran, J Craig Cohen, Janet E Larson

**Affiliations:** 1 The Brady Laboratory, Section of Neonatology, Department of Pediatrics, Stony Brook University, School of Medicine, Stony Brook, New York, 11794, USA

## Abstract

One of the mechanisms by which adult disease can arise from a fetal origin is by in utero disruption of organogenesis. These studies were designed to examine respiratory function changes in aging rats following transient disruption of lung growth at 16 days gestation. Fetuses were treated in utero with a replication deficient adenovirus containing the cystic fibrosis conductance transmembrane regulator (CFTR) gene fragment cloned in the anti-sense direction. The in utero-treated rats demonstrated abnormal lung function beginning as early as 30 days of age and the pathology progressed as the animals aged. The pulmonary function abnormalities included decreased static compliance as well as increased conducting airway resistance, tissue damping, and elastance. Pressure volume (PV) curves demonstrated a slower early rise to volume and air trapping at end-expiration. The alterations of pulmonary function correlated with lung structural changes determined by morphometric analysis. These studies demonstrate how transient disruption of lung organogensis by single gene interference can result in progressive change in lung function and structure. They illustrate how an adult onset disease can arise from subtle changes in gene expression during fetal development.

## Background

The diseases that result from prematurity often occur acutely in the perinatal period and are the result of an undeveloped organ exposed to the extra uterine environment. However, as survival of the acute perinatal period increases in these infants, observations have been made of an increased incidence of late or adult onset diseases in this population. These adult diseases include diabetes, obesity, cardiovascular disease, and asthma [1-4] and demonstrate how changes in the fetal environment can have a profound effect on physiology into the adult.

Lung organogenesis is in part dependent upon stretch-induced differentiation via contraction of the embryonic airway smooth muscle [5-7]. One protein recently shown by this laboratory to modify stretch induced lung organogenesis is the cystic fibrosis transmembrane conductance regulator protein or CFTR [8]. Multiple independent lines of evidence have suggested that CFTR is involved in lung development (for reviews see [1,9]). Recently, this laboratory demonstrated that in utero CFTR expression levels regulate Wnt/β-catenin signaling [10] through the parathyroid hormone related peptide (PTHrP) as demonstrated in the Troday-Rehan model for stretch-induced differentiation of the lung [11-15].

This laboratory developed the technique of in utero gene transfer into the pulmonary and intestinal epithelium using low dose adenoviruses [16-19]. In subsequent papers we and others have demonstrated that this method completely bypasses the inflammatory response normally seen in virus mediated gene transfer if performed with a low dose and at the proper developmental stage in mice, rats, and nonhuman primates [10,16,20-27]. In addition, it was demonstrated previously with both C-MYC and CFTR that gene function can be transiently inhibited by the in utero infection of the lung and intestines with an adenovirus carrying an antisense gene construct. This process results in an approximate 50% reduction in gene expression [10,24,25]. This method of transient in utero knockout was subsequently validated independently by traditional transgenic mouse technology when the role of Wnt/Myc signaling in gut development was confirmed [28].

The use of adenovirus transferred genes to the developing epithelium, called transient in utero knockout (TIUKO), was used previously with antisense CFTR and resulted in altered lung structure, constitutive inflammation, and increased airway reactivity in young adult rats [29]. These results suggested that a transient change in expression of a single gene during development could disrupt a developmental cascade and permanently change lung structure and function. Given the role of stretch induced differentiation in lung growth and development with the participation of CFTR in stretch induced regulation of Wnt/β-catenin signaling, transient alteration of CFTR can be equated with transient modification of stretch.

In this study, the TIUKO CFTR method was again used to interfere with stretch-induced lung organogenesis in the fetal rat. Lung structure and function were examined to determine if transient changes in a single fetal gene involved in mechanicosensory differentiation could result in progressive pathology in an aging lung.

## Methods

### In-utero gene transfer

An adenovirus carrying anti-sense CFTR (ASCFTR) gene fragment was constructed as previously described[25]. In utero gene transfer was performed at 16 days gestation using a recombinant adenovirus carrying either the ASCFTR or the control genes EGFP/LacZ. Both viruses used a CMV promoter for transgene expression. Timed-pregnant Sprague-Dawley rats were induced (5%) and sedated (2%) with inhaled isoflurane. The uterine horns were exposed by midline laparotomy and the individual amniotic sacs were exposed and externalized. Each individual amniotic sac was injected with a fine (27 gauge), needle containing adenoviral particles in Dulbecco's Minimal Essential medium at 10% of the amniotic fluid volume. The average final concentration of adenovirus was 10^8 ^pfu/ml of amniotic fluid. Prior studies showed this to be an efficient method of intrauterine gene transfer to the pulmonary epithelium [17]. Control rats underwent an identical surgical procedure but were injected with adenovirus carrying either EGFP or LacZ reporter genes. The mothers were allowed to deliver normally and the rat pups were raised under standard conditions in unfiltered cages to more closely replicate normal environmental exposures up to 18 months of age. The animals were analyzed serially at various time points up until 18 months of age. Routine monitoring of health by the vivarium staff did not reveal any evidence of chronic infections in either control or treated animals.

### Respiratory Function Testing

Animals undergoing pulmonary function testing were anesthetized with intra-peritoneal pentobarbital at a dose of 90 mg/kg. Anesthetic effect was monitored by tail pinch. Animals then underwent tracheotomy with a secured metal cannula and were connected to a flexiVent (SCIREC, Montreal, Canada) computer-controlled small animal ventilator. The animals were ventilated in a quasi-sinusoidal fashion at a rate of 150 breaths/min with an I:E ratio of 66.67%. Maximum peak inspiratory pressure was set at 30 cm of water. Cylinder piston displacement was set to provide a tidal volume of 10 ml/kg when gas compression was taken into account. Positive end-expiratory pressure (PEEP) was controlled by submerging the expiratory limb from the ventilator into a water trap. The animals were allowed five minutes to adjust to the ventilator at a PEEP of 3 cmH_2_O and then were paralyzed with an intraperitoneal injection of pancuronium bromide (0.5 mg/kg). Paralytics were required to completely inhibit any respiratory activity that would interfere with respiratory function testing. All animal protocols were approved by the institutional animal care and use committee.

### Respiratory mechanics

Automated respiratory function testing was performed using the flexivent ventilator. After cessation of spontaneous respiration, PEEP was set to 0 cm water and the rat was ventilated for 1 minute to equilibrate. Mechanical ventilation was interrupted and the animal expired against the set PEEP for 1 second. Dynamic PV curves were then determined. After renewed ventilation for 1 minute to re-equilibrate, an 8 second broad-band petrubation signal consisting of 18 equally spaced superimposed sine waves with frequencies ranging from 0.25 Hz to 19.625 Hz was applied to the lungs with the flexivent ventilator. Correction for mechanical characteristics of the ventilator circuit was made using dynamic callibration data. This was obtained by applying volume pertubations through the circuit both open and closed to the atmosphere prior to connection of animals to the ventilator. The ventilator was recalibrated between each animal. All measurements were made in triplicate and were repeated at PEEP of 3 and 6 cm of water after 1 minute of ventilation at each new PEEP to equilibrate.

Pulmonary impedance measurement was interpreted of in terms of the constant phase model [30]. Airway resistance (*Raw*) is a frequency independent Newtonian resistance reflecting the conducting airways [31]; G characterizes tissue damping; and H characterizes tissue stiffness (elastance). We also calculated hysteresivity (eta = G/H), which increases when regional heterogeneities develop in the lung [32]. We corrected for lung size using lung weight normalization for each animal. The forced oscillation technique described above has been used by other authors to perform respiratory function testing on both animals and human patients [33].

### Pressure-Volume Curves

Equal numbers of both treatment and control animals were analyzed. Dynamic pressure-volume curves were determined by inflating the lungs to a maximum pressure of 30 cm H_2_) abd allowing passive exhalation using the computer controlled Flexivent ventilator for measuring volume and pressure. All measurements were performed in triplicate. Individual results from each animal were compiled. Averages and standard deviations for each level of PEEP were determined. Two way ANOVA were performed on the data and results were graphed. PV curves were normalized by dividing volume by total lung compacity and graphed in Graphpad Prism 5.

### Histochemistry and Collagen Analysis

Masson's trichrome stain was performed (Sigma Chemical Co) on tissues fixed in methanol-free, 4% buffered paraformaldehyde. A blinded investigator captured images of trichrome stained lung tissue from 18 month old animals at a final magnification of 40×. Collagen content was determined by pixel count using Adobe Photoshop software [25,34].

### Morphometry

Animals undergoing morphometric analysis did not undergo respiratory function testing in order to preserve tissue integrity. Tissues were coded and identified by a number that each animal received at the time of sacrifice. This number was used for identification of all histology samples and served to blind the individuals performing morphometric analysis. The trachea was cannulated and the lungs were inflated at a constant pressure at 20 cm H_2_O for 24 hours in methanol-free 4% buffered paraformaldehyde. Lungs that did not maintain constant inflation were eliminated from the analysis. Sections from the upper, middle and lower left lung were embedded separately in paraffin for individual analysis [35].

Images from each entire section were captured at a final magnification of × 25 for point-counting morphometry. Volume densities of airway, parenchyma and vessels were estimated using a lattice of 121 test points. Parenchyma was defined as the gas-exchanging compartment that contained the alveoli and ducts. Airways consisted of conducting airways to the level of the terminal bronchioles.

In addition, 20 images of parenchyma from each section were captured at × 400 final magnification. Volume densities of airspace wall, airspace and inter-airspace wall difference were determined from these images. Inter-airspace wall difference (mean linear intercept (Lm)) was determined by counting the number of intercepts of a line of known length.

Two blinded investigators (in addition to the individual who captured the images) performed morphometry using the identification numbers with treatment groups unidentified.

### Statistical Analysis

Respiratory function testing and airway reactivity were analyzed using both paired t-test and ANOVA (GraphPad software). Following morphometric analysis, the upper, middle, and lower lobes were analyzed separately. No significant differences were attributed to specific lobes; therefore, morphometric data from lungs were pooled within each treatment group and age. Tissue volume proportion and collagen content were compared between the control and experimental groups using two-tailed t-test (GraphPad software). A p < 0.05 was considered statistically significant. All values are presented as mean ± standard error of mean.

## Results

### Adult airway histopatholgy following transient in utero gene intereference

Previous studies performed in this laboratory demonstrated an increase in collagen surrounding the airways at 100 days of age following in utero gene transfer of ASCFTR [25]. To determine the affects of aging on these airway changes, animals were examined at 18 months of age following fetal treatment. Fetuses treated at 16 days gestation with recombinant adenovirus carrying the ASCFTR were compared to control animals that had received AdCMVlacZ at the same gestation. These animals were examined after they were raised under standard conditions in unfiltered cages following normal delivery. The level of CFTR inhibition of coharts of the animals used in this study was documented and previously published [25]. CFTR expression was found to be reduced by approximately 50%.

**Figure 1:**
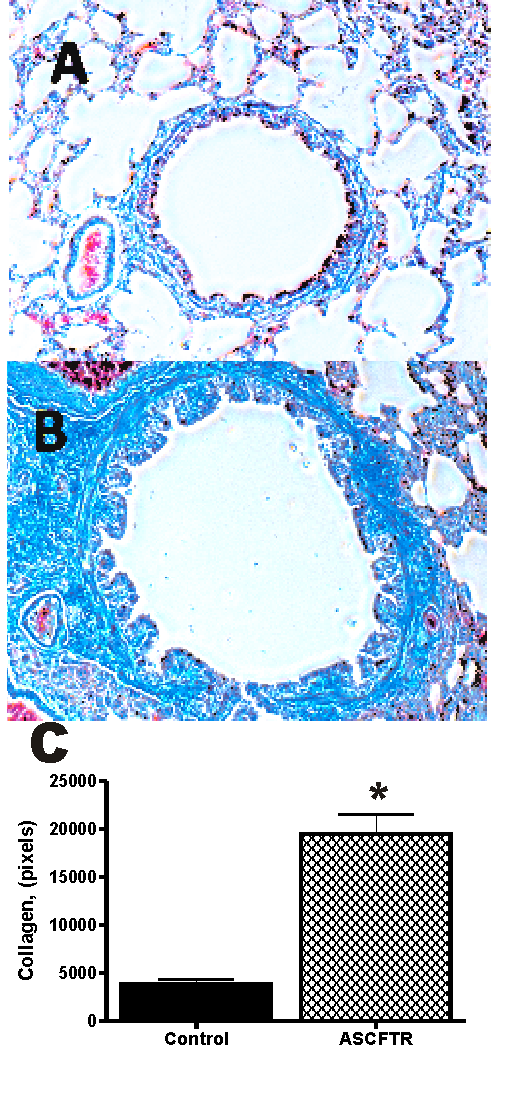
**Trichrome staining demonstrating increased collagen at 18 months of age following in-utero gene transfer**. (A) Airway of an 18 month old animal following injection at 16 days gestation with AdCMVlacZ (control). (B) Airway of an 18-month-old animal following injection at 16 days gestation with ASCFTR. (C) Pixel analysis of collagen content. *p < 0.0001.

Trichrome staining was performed on lung sections from both control and TIUKO CFTR animals at 18 months of age. Previous work in this lab demonstrated fibrosis in TIUKO CFTR animals at 100 days of age[25]. As shown in Figure 1, increased fibrosis was observed (demonstrated by blue stain) in animals in which lung organogenesis had been transiently disrupted with ASCFTR (panel B) as compared to the reporter gene, control, treated animals (panel A). Thus, the fibrosis observed previously at 100 days of age persisted into late adulthood. Photomicrographic quantitation via pixel counts of images (Figure 1, panel C) showed that a highly significant (p < 0.0001) increase in collagen content in the treated rat lungs as compared to the control group. There was no significant difference in the control pixel counts between 100 days (8) and 18 months demonstrating no fibrosis due to adenovirus vector. The collagen content in the ASCFTR treated lungs at 18 months was approximately 4-fold increase over controls as compared to a 1.7-fold increase over controls in the 100 day old animals [25]. Thus, the fibrotic lung histopathology in adult rats following TIUKO CFTR appeared to be progressive.

### Altered pulmonary mechanics in TIUKO CFTR rats at 18 months of age

Previous studies demonstrated altered airway reactivity and inflammatory changes in TIUKO CFTR animals as young adults [29]. These changes were shown to be unrelated to the gene therapy procedure as they were not observed in any of the previous publications by this and other laboratories using the in utero gene theapy method [10,16,20-27]. Given the airway histopathology observed at 18 months of age (Figure 1), one would expect persistantly altered respiratory mechanics in the lungs of the animals as they aged.

**Figure 2:**
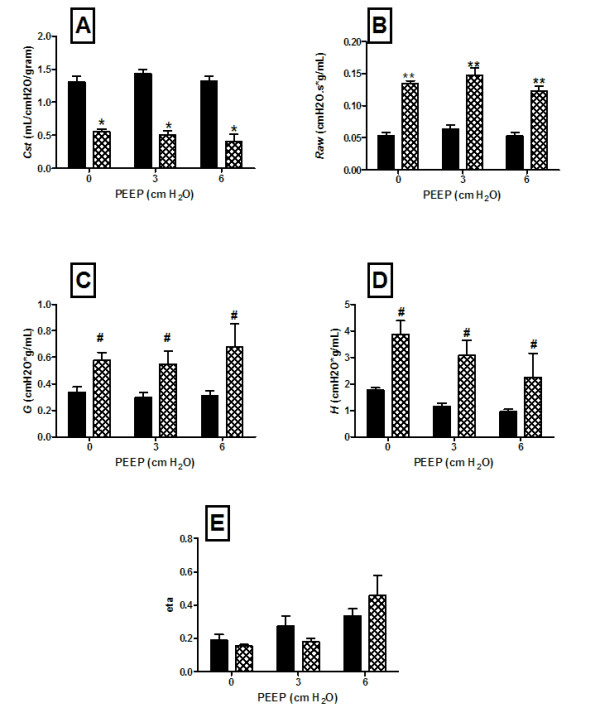
**Respiratory function at 18 months of age following in utero gene transfer at 16 days gestation**. Respiratory function in 18 month old animals following treatment at 16 days gestation with replication deficient adenovirus containing eGFP, (control, solid bars), or anti-sense CFTR gene fragment, (ASCFTR, crossed bars). (A) static compliance. (B), conducting airway resistance, (*Raw*). (C) tissue damping. (D) elastance. (E) hysteresivity, (eta). Four animals are included in each data point. All data were obtained in triplicate for each animal. Error bars are ± standard error of mean. * p < 0.005; ** p < 0.004; # p < 0.02.

Respiratory function tests were performed on 18 month old adult animals following ASCFTR treatment at 16 days gestation. A significant decrease in static compliance (Cst) was noted in the TIUKO rats; these results were consistent across all levels of PEEP (Figure 2, panel A). The decrease in static compliance was consistent with the increase in collagen content noted in the conducting airways of the TIUKO rats at the same age (Figure 1, panel B).

The constant-phase model analysis demonstrated a significant increase in conducting airway resistance (*Raw*) at all levels of PEEP (Figure 2, Panel B). In addition, there was a signficant increase in tissue damping (Figure 2, Panel C) which reflected altered tissue resistance. The independently determined constant phase model elastance (H) was significantly increased in the treatment group at all levels of PEEP, (Figure 2, panel D). Hysteresivity, (eta), was decreased at PEEP of 0 and 3 cm water but was not significantly increased at PEEP of 6, (Figure 2, Panel E).

**Figure 3:**
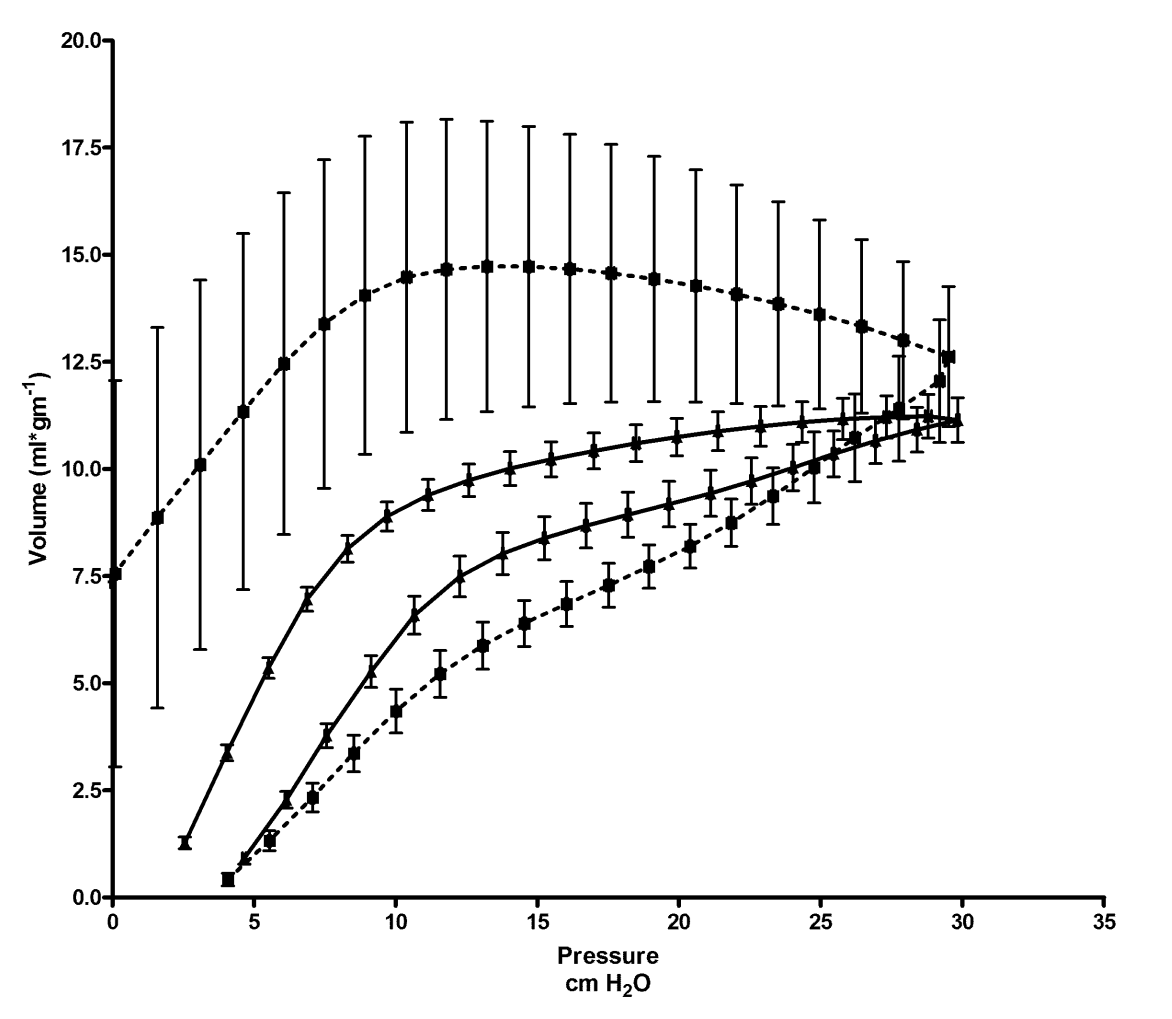
**Changes in PV curves at 18 months of age following in-utero gene transfer at 16 days gestation**. Respiratory function in 18 month old animals following treatment at 16 days gestation with replication deficient adenovirus containing eGFP, (control, solid lines), or anti-sense CFTR gene fragment, (ASCFTR, dashed lines). Four animals are included in each data point. PV curves were obtained in triplicate for each animal. All data is presented as mean ± SEM. p < 0.0001.

Pressure-volume (PV) curves demonstrated the requirement for higher pressures to inflate the lungs in treated rats during the early phase of the respiratory cycle (Figure 3). In addition, air trapping was noted as the PV loop did not return to baseline volume at the end of exhalation. Increased variability during the expiratory phase of the respiratory cycle was noted as was hyperinflation.

### Decreased airway density in TIUKO CFTR rats at 18 months of age

**Figure 4:**
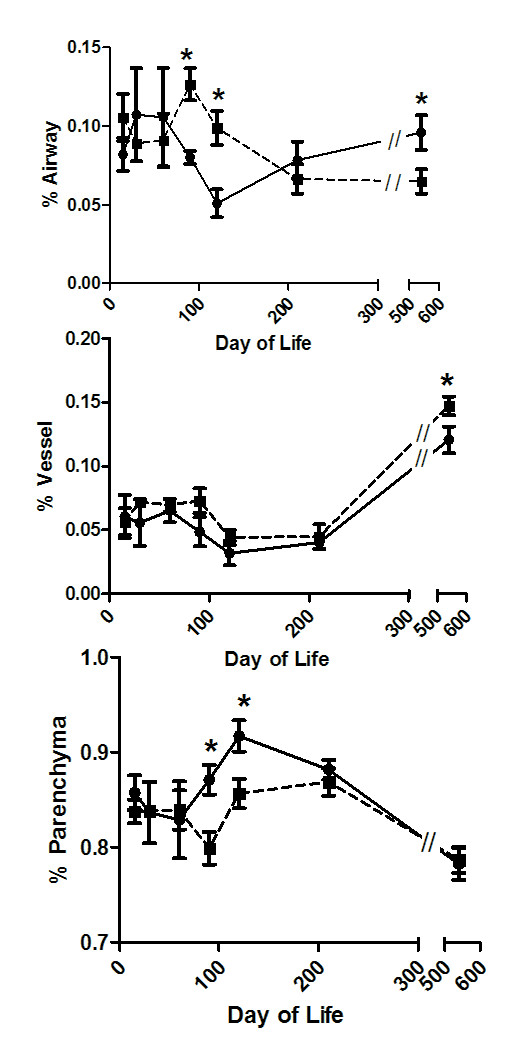
**Morphometric analysis of lung structure with age following in utero gene transfer at 16 days gestation**. Amniotic sacs were injected at 16 days gestation with replication deficient adenovirus containing either EGPF, (Control, solid lines), or anti-sense CFTR gene fragment, (Antisense, dashed lines). Morphometric analysis was performed on animals at 2, 4, 7, and 18 months of age. Volume proportions of airways (A), blood vessels (B), and parenchyma (C) are presented as mean ± SEM. *p < 0.05.

In addition to the increased collagen in the airways of the TIUKO CFTR animals at 18 months of age (Figure 1), the volume proportion of airways in the lungs was decreased in the TIUKO CFTR animals as compared to the controls (Figure 4, Panel A). The decreased airway density in the lungs of these animals may have also contributed to the increased conducting airway resistance that their pulmonary function testing demonstrated. In contrast, the volume proportion of blood vessels was increased at this age in the ASCFTR treated animals (Figure 4, Panel B).

### Alterations in parenchyma in TIUKO CFTR rats during adolescence

Static compliance, (Cst), was significantly decreased in adolescent animals as compared to their age-matched controls while *Raw *was unchanged during the same time period. In addition, elastance was signifcantly increased in TIUKO CFTR animals as compared to controls at 17 days of age and tissue damping was significantly increased in the ASCFTR group at 17 days of age. These changes suggested differences in parenchyma.

The parenchyma was examined closely at 400× final magnification with point counting. While the volume proportion of airways, vessels and parenchyma were unchanged in the adolescent animals, quantitative evaluation of the parenchyma demonstrated marked differences in these young animals (Table 1). When the parenchyma was specifically examined, there was an increase in volume density of airspace wall and a decrease in volume density of airspace. The complexity of the lung, suggested by Lm, was decreased. While these changes were highly significant in the adolescent animals, significance was lost while the animals aged.

**Table 1:** Effect of in utero ASCFTR on lung parenchyma.

Age (days)	Group	% Parenchyma	% Airspace	A/P	Lm
13	Control (eGFP)	0.18956612	0.712707	3.787066	18.8125
13	ASCFTR	0.24464738	0.673135	2.806436	20.99217
		p = .003	p = .03	p = .004	p = .012
					
60	Control (eGFP)	0.12055785	0.773347	6.508026	19.15
60	ASCFTR	0.12007576	0.778168	6.862095	20.2
		NS	NS	NS	NS
					
90	Control (eGFP)	0.19871534	0.790174	4.622078	21.62444
90	ASCFTR	0.21126822	0.765983	4.568619	19.96945
		NS	NS	NS	NS
					
120	Control (eGFP)	0.16403409	0.850143	5.70228	18.405
120	ASCFTR	0.16472577	0.880894	5.84488	18.14
		NS	NS	NS	NS
					
210	Control (eGFP)	0.14146006	0.802755	6.003154	18.54375
210	ASCFTR	0.1651343	0.771823	4.979818	18.59688
		NS	NS	NS	NS

Detailed morphometric examination of the parenchyma at each age was performed by point counting at × 400 final magnification. Twenty images were examined from upper, middle and lower sections of each left lung following fixed-inflation. Volume densities of airspace wall, airspace and inter-airspace wall difference were determined by point counting. Inter-airspace wall difference (mean linear intercept (Lm)) was determined by counting the number of intercepts of a line of known length.

### Alterations in lung structural changes in TIUKO CFTR rats as a function of age

To determine if pulmonary structural alterations and tissue remodeling reflected the altered pulmonary mechanics, morphometric analysis was performed on the lungs of the animals as they aged. As with the pulmonary mechanics, the young ASCFTR adults enjoyed periods of relative structural normality and morphometric analysis of young adults did not show any differences in the volume densities of the airways, parenchyma or vessels. However, alterations occurred as the animals aged (Figure 4).

In the control animals, the volume proportion of parenchyma was highest in young adulthood. With age the density of airways and vessels increased and the volume proportion of parenchyma decreased (Figure 4, solid lines).

During their adolescence, both the control and TIUKO CFTR animals showed wide variance in their volume densities. During that time the ASCFTR adolescent lung structure did not vary significantly from their aged-matched controls. After 60 days of age there was an increase in the volume proportion of airways in young adult animals treated with ASCFTR as compared to control animals (Figure 4, panel A); these differences remained through 90–120 days of age and corresponded to a significant decrease in the volume proportion of parenchyma at the same age (Figure 4, panel C). This differed markedly from the statistically signifcant decrease in the airway density of the old adults treated in utero with ASCFTR. At 210 days of age the the volume proportion in the airways and parencyma did not vary significantly from the conrol group. After 210 days of the control group increased its airway density while the ASCFTR group did not. This resulted in a signficant decrease in airway density as compared to controls at 18 months of age (Figure 4, Panel A). These data are futher evidence of progressive disease throughout adulthood despite the transient nature of the ASCFTR treatment.

The volume proportion of blood vessels was consistently increased throughout adulthood in the TIUKO CFTR animals, however this difference only reached significance at 18 months of age (Figure 4, Panel B).

### Altered pulmonary mechanics in TIUKO CFTR rats as a function of age

**Figure 5:**
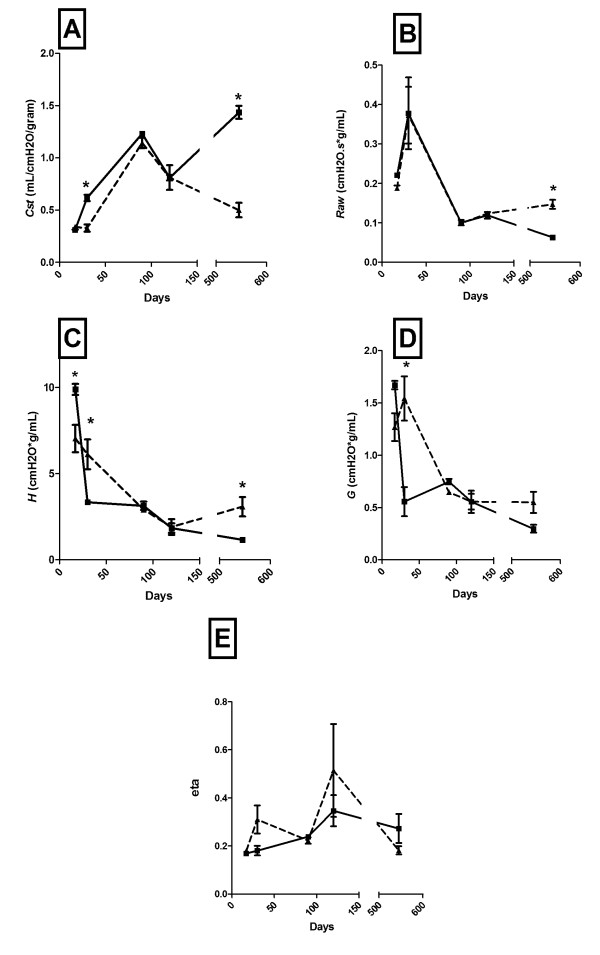
**Respiratory function with age following in-utero gene transfer at 16 days gestation**. Respiratory function testing at 17, 30, 90, 120 days and 18 months, (PEEP 3 shown) following in utero treatment with an adenovirus containing either EGFP, (control, solid lines), or anti-sense CFTR gene fragment, (ASCFTR, dashed lines). (A) static compliance (*Cst*). (B) conducting airway resistance, (*Raw*). (C) elastance (D) tissue damping. (E) hysteresivity, (eta). All data were obtained in triplicate. Eight animals are included in each data point. Error bars are ± standard error of mean. Results at PEEP of 0 and 6 were similar, (data not shown). *p < 0.05.

Respiratory function was examined in the ASCFTR animals at various timepoints up to 18 months of age (17, 30, 90, 120, and 540 days of age). Changes of respiratory mechanics over time are presented in Figure 5.

Static complinace, (Cst), was significantly decreased in adolescent ASCFTR treated animals as compared to their age-matched controls (Figure 5, Panel A). However, these values normalized as the animals reached young adulthood and at 90–120 days of age the static compliance in the TIUKO CFTR animals did not vary significantly from the control animals. However, as the animals aged, the TIUKO CFTR adults demonstrated significant decreases in their static compliance as compared to their control counterparts.

In contrast, conducting airway resistance, (*Raw*), was initially normal in the young TIUKO CFTR animals as compared to their age-matched controls. The large differences in airway resistance appeared only as the animals became aged into late adulthood (Figure 5, Panel B).

Elastance, (*H*), demonstrated the same bimodal pattern as *Cst*. At 30 days of age elastance was signifcantly increased in TIUKO CFTR animals as compared to controls. These values normalized and there was loss of significance during young adulthood (90–120 days) with a return to a significant increase by 18 months of age (Figure 5, Panel C). A decrease in elastance is a marker of normal lung development in the adolescent and it is known to decrease steadily until 17 days of age in the Sprague-Dawley rat [36]. The elevation of elastance through 30 days of age was consistent with a delay in maturation in the TIUKO CFTR animals. Although the young adults were able to exhibit near normal functions during young adulthood (similar to Cst and *Raw*), this parameter also began to deteriorate as the animals aged.

Tissue damping, (*G*), was significantly increased in the ASCFTR group at 30 days of age. By 90 days of age there was loss of significance (Figure 5, Panel D). Hyteresivity, (eta), showed marked variability as the animals aged (Figure 5, Panel E). By 18 months of age it was significantly decreased at PEEP of 0 and 3 cm H_2_O.

## Discussion

During normal development the fetus is exposed to numerous transient insults that can affect organogenesis. Using standard methods for manipulating gene expression such as transgenic mice with and without inducible promoters it is impossible to depress gene expression in a small number of cells and then have that gene recover due to normal cell turnover or expansion. The transient in utero gene transfer system used in this study is the only method affecting a specific gene. In addition, the stoichiometry of this method results in disruption of only a very small number of cells. A total of <10^7 ^infectious units are delivered to the entire fetus. Given the distribution of virus to the lung, intestines, skin, and amnion [16,17,27] less than 10^6 ^cells are transfected with the transgene in the lung.

Until the recognition of the role of CFTR in stretch-induced regulation of muscle contractions and Wnt/β-catenin signaling [8,10] it was difficult to understand how affecting such a small number of cells could have significant effects on lung structure and function. Because the process of stretch-induced differentiation is a global process, small changes would be amplified by altered expression of genes such as CFTR or Rho kinase[37].

Transient in utero knockout of CFTR resulted in a pattern of evolving respiratory function and structure with age. During their adolescence the animals treated in utero with ASCFTR demonstrated mechanical and histologic evidence of parenchymal immaturity. In young adulthood the animals enjoyed a period of relative health followed by progressive disease culminating in significant mechanical and histologic disease at 18 months of age. That these results were specific to the transient inhibition of CFTR in utero can be seen with the normal lungs in control animals treated with adenovirus reporter, thus it is not due to in utero adenovirus-specific lung response. In addition, previous studies with adenovirus mediated over expression of CFTR in which lung growth and development was actually stimulated and enhanced pulmonary functions observed [20,21,26]

Adolescent animals following in utero treatment with ASCFTR showed signficant alterations in respiratory function compared to their aged-matched controls. Specifically, tissue damping and elastance were elevated at 30 days, while compliance was decreased. In addition, morphometric analysis of the parenchyma at this age demonstrated an increase in volume density of the airspace wall, a decrease in volume density of airspace and decreased complexity. These findings deviate from the normal developmental pattern of respiratory function as described by Broussard et al. In the Sprague-Dawley rat tissue damping and elastance declines through 17 days of age until reaching an equilibrium. This coincides with alveolarization and thinning of the interstitium [36]. The delay in the normal decline in tissue damping and elastance in the TIUKO CFTR animals is consistent with a delay in lung development. This theory is supported by other work in this laboratory demonstrating a CFTR-dependant cascade that affects cytoskeletal tension during lung organogenesis [1,8].

The TIUKO CFTR animals enjoyed a period of normal pulmonary mechanics in young adulthood (90–120 days of age). In contrast, the volume density of their airways was increased by morphometric analysis at the same age. This is not dissimilar to findings in young children with CF. Recently, high-resolution computed tomography imaging has demonstrated that infants with CF have more dilated airways with thicker walls in the absence of abnormal pulmonary function [38]. At 210 days of age the volume proportion of the airways and parencyma did not vary significantly from the conrol group.; had morphometric analysis been done only at that time point no differences would have been noted.

Late adulthood following in utero ASCFTR treated was associated with a progressive decline in respiratory function. By 18 months of age there were signficant differences in both the structure and function of the lungs of the ASCFTR animals as compared to their aged-matched controls. The lungs demonstrated decreased Cst and increased H. Conducting airway resistance was increased at this time. Increased tissue damping suggested changes in the parenchymal tissue and the decrease in hysteresivity reflected inhomogeneity of the lungs. The loss of significance in eta at higher levels of PEEP suggested that this inhomogeneity is due to both focal fibrosis as well as surfactant system dysfunction.

The PV curves in the animals at 18 months of age demonstrated several abnormalities. Greater pressure required to inflate the lungs as well as air trapping at end expiration were noted. In addition, a large amount of variability was noted on the exhalation phase of the respiratory cycle. This is the passive phase on the ventialtor, completely dependent on lung properties. The variability is due to the different responses of each animal to environmental conditions and represents the inhomogenity of the disease process. Adult patients with cystic fibrosis often demonstrate air trapping on pulmonary function testing similar to that found in our ASCFTR group [39-43]. They also demonstrate hyperinflation and decreased compliance as was demonstrated in our ASCFTR animals [40-44]. Our results are consistant with these characteristics of lung function found in the adult cystic fibrosis patients population.

In addition, there were structural changes in the lungs by 18 months of age in the TIUKO CFTR group. The older animals demonstrated a decrease in volume density of airways and an increase in volume density of blood vessels. There was an increase in collagen content in these lungs. These findings are consistent with a pattern of tissue destruction and remodeling.

Chronic inflammation in the lungs is a hallmark of cystic fibrosis. It is still debated wether this early chronic inflammatory state exists as a primary component of cystic fibrosis or if persistent infection causes this inflammation. Regardless of the cause, this chronic inflammation leads to obstructive lung disease and tissue destruction. This results in bronchiectasis and respiratory failure over time. While inflammation was not addressed in this paper, previous work in this laboratory demonstrated a constitutive pro-inflammatory state in the TIUKO CFTR treated animals [29]. A pro-inflammatory state in CFTR deficiency has been demonstrated by others [45-49].

This work demonstrates that transeint disruption of stretch-induced organogenesis can result in progress lung disease due to disruption of organogenesis. Interestingly, some aspects of CF pathology may originate in fetal life from the total absence of CFTR. As migh be expected CF pathology would be much greater than that observed in this transient model.

The fetal origin of adult disease has been recognized and debated for nearly two decades with growing evidence in support [50-56]. Recent work in this laboratory describes cystic fibrosis as a "Peter Pan Disease" where the lung is immature at birth and never "grows up" [1]. This immaturity leads to the progressive disease process observed in our animal model and possibly has some contribution to humans with CF. There is a critical period in fetal development where CFTR expression is required for normal lung development. Alteration of CFTR expression during this time period results in an immature lung by alteration of stretch-induced organogenesis. It also results in an altered inflammatory state with a shift toward chronic low-grade inflammation. Despite recovery of normal CFTR expression subsequent to this critical developmental period the lung never recovers.

## Competing interests

The authors declare that they have no competing interests.

## Authors' contributions

JH performed the pulmonary function testing and morphometry with the assistance of JCC and JL. EK preformed the in utero gene therapy and virus preparation. AC performed the collagen analysisn. JCC and JL were responsible for the overall design and exceution of this project.
